# Acute Effects of External, Internal, and Holistic Attentional Focus on Javelin-Throwing Performance in Novices: The Role of Visual Information

**DOI:** 10.3390/jfmk11030329

**Published:** 2026-08-24

**Authors:** Ayoub Asadi, Somayeh Bahrami, Mostafa Bahrami, Esmaeel Saemi

**Affiliations:** 1 Department of Kinesiology, Iowa State University, Ames, IA 50011, USA; asadi68@iastate.edu; 2 Department of Sports Physiology, Faculty of Humanities, Lorestan University, Khorramabad 6815144316, Iran; somayeh.bahrami7@yahoo.com (S.B.); bahrami.ms@lu.ac.ir (M.B.); 3 Department of Motor Behavior and Sport Psychology, Faculty of Sport Sciences, Shahid Chamran University of Ahvaz, Ahvaz 6135783151, Iran

**Keywords:** attention, motor skills, athletic performance, vision

## Abstract

**Background and Objectives**: Sports performance is influenced by a variety of perceptual, cognitive, and motor factors. A better understanding of these factors can help coaches optimize training and improve athletic performance. The present study examined the acute effects of internal, external, and holistic attentional focus instructions on javelin-throwing performance under different visual conditions in novice performers. **Methods**: Twenty-four university students (12 females and 12 males; mean age: 21.50 ± 1.47 years) with no prior javelin-throwing experience completed 30 throws under six experimental conditions (5 throws per condition) based on three attentional focus instructions (internal, external, and holistic) and two visual conditions (vision-present and vision-absent) using a within-subject repeated-measures design. Throwing distance was measured as the primary performance outcome. **Results**: The results revealed significant main effects of both vision (F_(1, 23)_ = 14.49, *p* = 0.001, ηp^2^ = 0.38) and attentional focus (F_(2, 46)_ = 5.73, *p* = 0.006, ηp^2^ = 0.20). Overall throwing performance was superior when visual information was available compared to when vision was occluded (*p* = 0.001). In addition, both external and holistic focus conditions produced significantly greater throwing distances than the internal focus condition (*p* = 0.03; *p* = 0.04, respectively), with no significant differences observed between external and holistic focus (*p* > 0.05). No significant interaction was found between attentional focus and vision (F(_2, 46_) = 0.74, *p* = 0.48, ηp^2^ = 0.03), indicating that the effect of attentional focus did not significantly differ between the visual conditions. **Conclusions**: The present findings extend the attentional focus literature to a complex, whole-body ballistic skill, demonstrating that the adoption of external and holistic attentional focus instructions resulted in greater throwing distances than the internal focus condition in novice javelin throwers.

## 1. Introduction

The performance of motor skills is influenced by various factors, including practice conditions, motivational influences, and instructional strategies [1,2]. Among these variables, attentional focus has consistently been investigated as a key determinant of motor performance and learning [3,4]. Additionally, verbal instructions that direct athletes’ attentional focus during skill execution play a crucial role in shaping motor performance [5]. Attentional focus in motor skills refers to the direction of a performer’s attention during movement execution, which can significantly influence motor performance and learning [4]. It is generally categorized into two types: internal focus, where attention is directed toward the body’s own movements, such as focusing on extending the knee; and external focus, where attention is directed toward the effects of the movement on the environment, such as focusing on a cone during jumping [6,7]. Research has shown that providing instructions that encourage an external focus rather than an internal focus enhances motor skill performance across the lifespan in children, adults, and older adults [4,8,9]. The constrained action hypothesis suggests that an external focus allows the motor system to function more automatically and execute skills more smoothly [10]. In contrast, directing attention internally disrupts this automatic control, leading to slower, less accurate, and weaker movements [11]. Studies across various gross motor skills, including javelin throwing [12], horizontal jumping [6,7,13,14,15], and sprinting [11], support the advantages of an external focus. However, despite numerous reports on the superiority of an external over an internal focus of attention [3,4], recent meta-analyses have suggested that publication bias may have influenced these findings. Consequently, the advantage of an external focus may depend on the characteristics and complexity of the motor task [16].

In recent years, holistic focus has emerged as a novel attentional strategy that directs performers’ attention toward the overall sensation and fluidity of the movement [17,18]. This approach has been proposed as an alternative to the traditional dichotomy between internal and external attentional focus, especially in complex motor skills where neither internal nor external cues seem ideal. According to Becker et al. [17], a holistic focus emphasizes the “general feeling” of movement, making it particularly useful for activities such as gymnastics or dance, where focusing on specific body parts or environmental effects is less intuitive [15]. Studies have demonstrated that a holistic focus can yield similar benefits to an external focus, often outperforming an internal focus in various tasks [7,15,17,18,19,20,21,22]. For example, Saemi et al. [7] showed that instructing novice athletes to adopt either an external or a holistic focus led to greater standing long jump distances than directing attention internally or providing no specific focus. Importantly, performance did not differ between the external and holistic focus conditions. Similarly, Noroozi et al. [15] showed the advantage of external and holistic focus instructions in standing long jump performance compared to internal focus and control conditions in skilled and novice female karate athletes.

Vision, as a primary source of sensory feedback, plays a crucial role in the planning, adjustment, and accuracy of motor skills [23]. Research has shown that vision may interact with attentional focus effects on motor performance [24]. In balance tasks, Becker and McNamara [24] demonstrated that the effectiveness of attentional focus strategies depends on visual availability: participants without visual occlusion exhibited lower root mean square error (RMSE) under an external focus, whereas those performing with visual occlusion showed lower RMSE under an internal focus. However, empirical evidence on motor skill tasks suggests that the effectiveness of attentional focus strategies, especially an external focus, may not be fully dependent on visual information [25,26,27,28]. For instance, Abdollahipour et al. [25] investigated whether vision mediates the benefits of an external focus of attention in a body projection task (vertical jump). Their results demonstrated that an external focus significantly enhanced jump height compared to internal focus and control conditions under both full-vision and no-vision conditions. Although overall performance was superior with full vision, no interaction between attentional focus and visual condition was observed, indicating that the benefits of an external focus operate independently of visual feedback in this task [25]. Similarly, Saemi et al. [27] showed that directing attention externally improved free-throw accuracy in skilled basketball players, even when visual information was occluded. Collectively, these findings suggest that while vision enhances overall performance, the advantages of external attentional focus can be preserved even in the absence of visual input.

Although traditional attentional focus research has used internal–external instructions [3,4], this dichotomy may oversimplify performers’ focus of attention during complex motor tasks. Recent evidence suggests that a holistic focus offers an alternative to binary classifications; however, its interaction with sensory constraints remains poorly understood. In particular, the role of visual information in moderating the effectiveness of external versus holistic attentional focus has received little empirical attention. External focus strategies are inherently oriented toward movement effects and may therefore depend more strongly on the availability of vision to guide performance. In contrast, a holistic focus emphasizes the overall coordination of the movement, and it may rely less on visual information; this feature could make holistic focus more robust when vision is absent. To date, however, no research has directly examined how external and holistic attentional focus strategies perform across different levels of visual availability.

The present study aimed to examine the effects of internal, external, and holistic attentional focus instructions on javelin-throwing performance under available and occluded vision. Based on prior research indicating that attentional focus benefits, particularly those associated with an external focus, can persist in the absence of vision [25,27], and the similar benefits observed between holistic attention and external attention [7,15,17,18,19,20,21,22], it was hypothesized that throwing distance would be greater under external and holistic attentional focus than under internal attentional focus. It was also hypothesized that throwing distance would be greater when visual information was available than when vision was occluded. Finally, it was hypothesized that the effect of attentional focus on throwing performance would differ between visual conditions, with holistic attentional focus showing a smaller reduction in throwing distance following visual occlusion than external attentional focus.

## 2. Method

### 2.1. Participants

In this counterbalanced within-participant repeated-measures experiment, 24 undergraduate students (12 males, 12 females; age range: 19–26 years; mean age: 21.50 ± 1.47 years) with normal vision were recruited from a physical education class using convenience sampling. The initial sample size was calculated using G*Power software (version 3.1.9.4). Based on an estimated interaction effect size between attentional focus and vision of ηp^2^ = 0.13 (f = 0.38) [25], an alpha level of 0.05, statistical power of 0.95, a 3 × 2 within-participant repeated-measures design, a correlation of 0.50 among repeated measures, and a nonsphericity correction (ε) of 1.0, a minimum sample size of 20 participants was estimated for the interaction between attentional focus and vision. To account for potential participant attrition and to maintain consistency with previous related studies [25], the final sample was increased to 24 participants. Before starting data collection, all participants provided written informed consent to participate in the study. Participants self-reported being right-handed and free from neurological, psychological, developmental, motor, or visual disorders, and none had prior experience in javelin throwing. All participants completed the study, and all throws were performed with the right hand. Any participant who did not meet the criteria was excluded from the study. The research project was designed based on the Declaration of Helsinki and was approved by the Research Ethics Committee of Shahid Chamran University of Ahvaz (IR.SCU.REC.1404.131).

### 2.2. Apparatus and Task

The javelin throw served as the motor task in this study, and throwing distance was measured as the primary outcome. All throws took place in the university athletics stadium [12]. Participants performed all throws using official tournament javelins (600 g for females and 800 g for males). The runway was defined by two parallel lines, 5 cm wide and 4 m apart (consistent with standard javelin specifications).

In addition, participants were instructed to release the javelin with the lead (left) foot positioned directly behind the throwing arc, which served as the reference line for distance measurement. The experimenters monitored each trial to verify the participant’s release position relative to the throwing arc. In the vision-occlusion conditions, participants initiated their throw based on their estimated approach length before visual occlusion, and, consequently, the release position could deviate from the throwing arc because the arc was not visually available at the moment of release. These release-position deviations from the throwing arc were recorded visually by the researchers based on the observed position of the participant during the throwing movement. For the final analysis, throwing distance was consistently calculated from the throwing arc to the first point of contact of the javelin with the ground for all six experimental conditions. Thus, the same reference point was used to define throwing distance across all conditions. Throwing distance was measured using a measuring tape with an accuracy of 1 cm. All measurements were performed by the same researcher.

To verify adherence to the attentional focus instructions, participants completed a manipulation check immediately after each five-throw block (i.e., each experimental condition). They rated the extent to which they followed the prescribed instruction on a 7-point Likert scale ranging from 1 (“not at all”) to 7 (“completely”) [29]. At the end of each visual condition, participants also reported which of the three attentional focus strategies (internal, external, or holistic) they preferred.

### 2.3. Procedure

Each participant completed a single testing session (approximately 90 min). First, participants completed demographic and health questionnaires. Then, they performed a standardized warm-up. After the warm-up, proper javelin-throwing technique was demonstrated and explained by a qualified coach. The instructions included the correct grip, carrying position, approach run, withdrawal, crossover steps, delivery, release, and follow-through. To ensure participant safety, the experimenters retrieved the javelins after each throw, and participants remained behind the throwing area until instructed to prepare for the next trial.

In the vision-occlusion conditions, participants viewed the throwing runway and target area before each trial. Immediately before initiating the approach run, the blindfold was applied and remained in place until the javelin had been released, after which it was removed by the experimenter. Thus, visual occlusion eliminated online visual information during movement execution but did not prevent participants from relying on previously viewed spatial information.

Across conditions, participants were instructed to maximize throwing distance while adhering to the prescribed attentional focus instructions. In the external focus (EF) condition, they were instructed as follows: “As you try to perform your best throw, try to mentally focus on the flight path of the javelin toward the target area.” In the holistic focus (HF) condition, they were instructed as follows: “As you try to perform your best throw, try to mentally focus on the smoothness and fluidity of the entire throwing movement.” In the internal focus (IF) condition, they were instructed as follows: “As you try to perform your best throw, try to mentally focus on the movements of your throwing arm during javelin release” [7]. Visual occlusion was carried out through a specialized blindfold that eliminated visual input during movement execution [27].

The experimental protocol consisted of 5 familiarization trials in each visual condition followed by 30 test throws (5 trials × 6 conditions) in a counterbalanced order (six sequences based on a balanced Latin square; four participants per sequence). During the familiarization phase, participants received initial instruction from a qualified coach regarding proper javelin-throwing technique. The coach provided standardized instructions on the throwing technique and its execution to each participant. Although participants were not required to meet a formal minimum technical-performance criterion before the experimental trials, corrective feedback was provided by the coach when necessary during familiarization to ensure that participants understood the instructions and could perform the technique safely. During the testing phase, however, no technical corrections, performance feedback, or verbal encouragement were provided. To control for fatigue effects, one-minute rest intervals were implemented between throws, and five-minute breaks were taken between different experimental conditions. Trials in which participants released the javelin beyond the throwing arc or threw outside the legal sector were repeated. The mean throwing distance of the five valid throws obtained in each experimental condition was analyzed, while invalid throws were repeated and were not included in the calculation of throwing distance. No participant withdrew from testing because of safety concerns or injury. Javelin-throwing performance was calculated as the mean distance achieved across the five throws for each participant in each experimental condition and was subsequently entered into the statistical analyses.

### 2.4. Data Analysis

To analyze throwing distance, a 3 attentional focus (internal, external, and holistic) × 2 vision availability (vision-present and vision-absent) RM-ANOVA was conducted to examine main effects and interaction effects. When significant main and interaction effects were detected, Bonferroni-adjusted post hoc comparisons were performed. The chi-square goodness-of-fit test was used to examine the distribution of participants’ preferences for the three attentional instructions separately within each visual condition. In addition, for each participant, the coefficient of variation (CV) was calculated as the standard deviation of the five valid throws divided by the participant’s mean throwing distance, multiplied by 100. The mean CV for each condition was then calculated by averaging the individual CV values across the 24 participants. Effect sizes were calculated using partial eta squared (ηp^2^) and interpreted according to Cohen’s [30] guidelines, with values of 0.01 (small), 0.06 (medium), and 0.14 (large). The normality of data distribution was examined using the Shapiro–Wilk test. All inferential statistical analyses were carried out in IBM SPSS Statistics (Version 24.0), at an alpha level of 0.05.

## 3. Results

The descriptive findings regarding participant characteristics showed that women had a mean age of 21.75 ± 1.66 years, mean height of 167.17 ± 8.17 cm, and mean weight of 62.67 ± 8.00 kg, whereas men had a mean age of 21.25 ± 1.29 years, mean height of 175.92 ± 5.99 cm, and mean weight of 71.00 ± 6.73 kg. Across all participants, the mean age was 21.50 ± 1.47 years, mean height was 171.54 ± 8.31 cm, and mean weight was 66.83 ± 8.39 kg. Regarding javelin-throwing performance and coefficients of variation (CV) across the six experimental conditions, the descriptive findings, including the mean, SD, 95% confidence interval, and mean CV (%), are reported in Table 1.

### 3.1. Javelin-Throwing Performance

**Figure 1:**
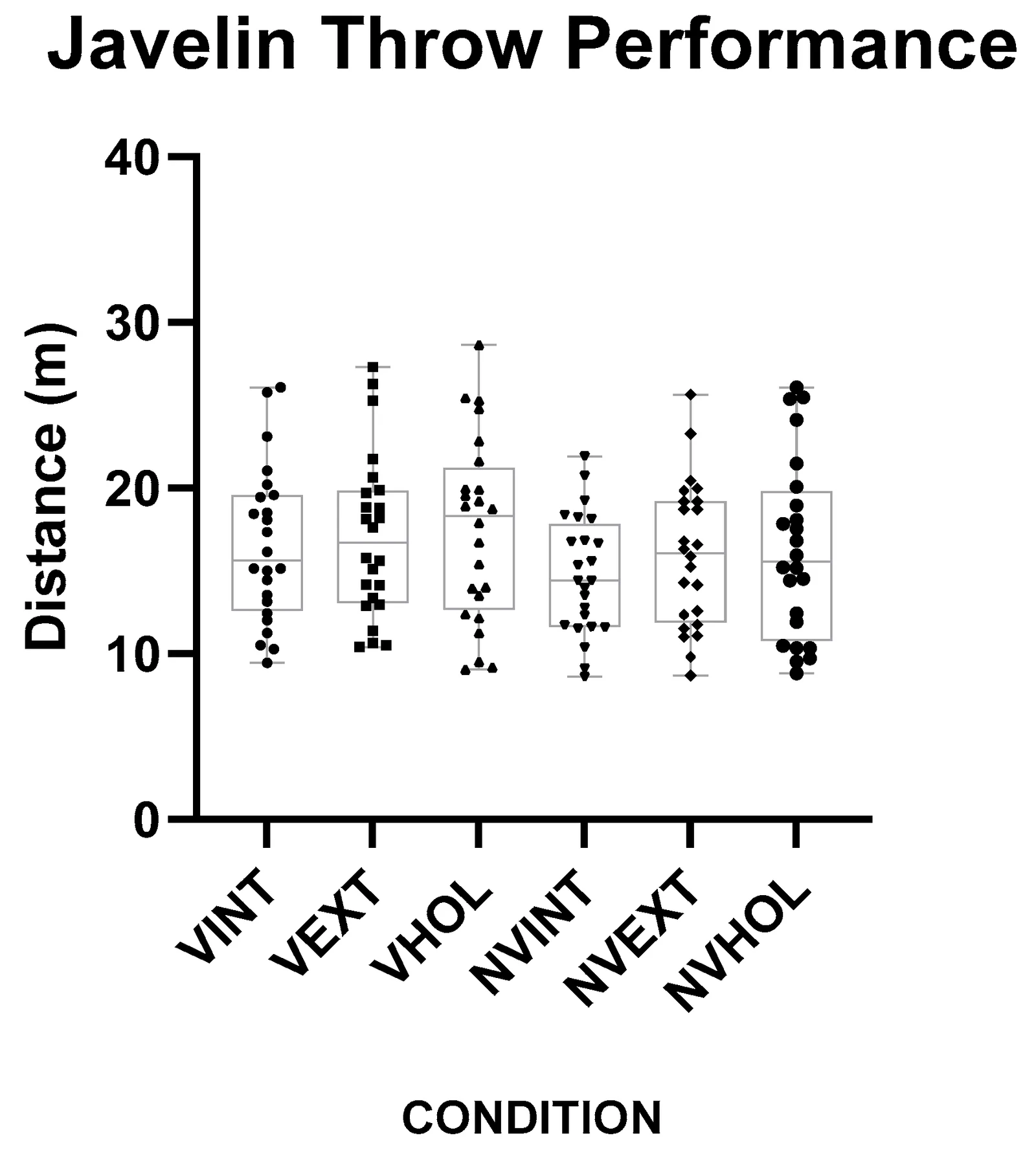
Javelin-throwing performance across the six experimental conditions. Box plots show the distribution of participant-level mean throwing distances (*n* = 24) in each condition, with individual points representing the mean distance across five throws for each participant. The boxes represent the interquartile range, and the horizontal lines within the boxes indicate the medians. VINT = internal attentional focus in the presence of vision; VEXT = external attentional focus in the presence of vision; VHOL = holistic attentional focus in the presence of vision; NVINT = internal attentional focus in the absence of vision; NVEXT = external attentional focus in the absence of vision; NVHOL = holistic attentional focus in the absence of vision.

The results of the 3 × 2 two-way repeated-measures ANOVA, with attentional focus (internal, external, and holistic) and vision (vision vs. no vision) as within-subject factors, conducted to compare different attentional as well as visual conditions, showed that the main effects of attentional conditions (F(2, 46) = 5.73, *p* = 0.006, ηp^2^ = 0.20) as well as visual conditions (F(1, 23) = 14.49, *p* = 0.001, ηp^2^ = 0.38) were significant, whereas the interaction effect was not statistically significant (F(2, 46) = 0.74, *p* = 0.48, ηp^2^ = 0.03). The results of the Bonferroni post hoc test revealed that participants threw significantly farther under the vision condition (17.02 m) compared to the no-vision condition (15.66 m) (95% CI = 0.62–2.10, *p* = 0.001; mean difference = 1.36 m; Figure 1).

The results also indicated that, when averaged across the two visual conditions, participants threw significantly farther under holistic focus (16.89 m) than under the internal focus condition (15.62 m; 95% CI = 0.004–2.53, *p* = 0.04; mean difference = 1.26 m). Similarly, when averaged across the two visual conditions, participants threw significantly farther under external focus (16.51 m) than under the internal focus condition (95% CI = 0.04–1.73, *p* = 0.03; mean difference = 0.89 m). The results also showed that no statistically significant difference was detected between external and holistic focus (95% CI = −1.18–0.42, *p* = 0.71; mean difference = −0.37 m; Figure 1).

### 3.2. Adherence to Attentional Focus

The results of responses to the question regarding the degree of adherence to applying the prescribed attentional focus after each attentional condition showed that participants focused their attention on the relevant cues under the VINT (4.95 ± 1.54), VEXT (5.66 ± 1.27), VHOL (4.83 ± 1.40), NVINT (5.04 ± 1.62), NVEXT (5.12 ± 1.48), and NVHOL (4.75 ± 1.89) conditions.

### 3.3. Attentional Focus Preference

To examine participants’ preference for applying attentional focus, after each visual or non-visual condition, individuals reported their preferred attentional focus. The results of the chi-square goodness-of-fit test indicated that after the vision condition, although most participants preferred external focus (13 participants; 54.2%) compared to the other attentional instructions, holistic focus (4 participants; 16.7%) and internal focus (7 participants; 29.2%), no significant difference was observed among them (χ^2^(2) = 5.25, *p* = 0.07).

After the no-vision condition, although most participants preferred external focus (11 participants; 45.8%) compared to the other attentional instructions, holistic focus (7 participants; 29.2%) and internal focus (6 participants; 25%), no significant difference was observed among them (χ^2^(2) = 1.75, *p* = 0.41).

## 4. Discussion

The present study examined the effects of internal, external, and holistic attentional focus instructions on javelin-throwing performance under available and occluded vision in novice performers. It was hypothesized that external and holistic focus instructions would result in superior performance compared with an internal focus when visual information was available and that this performance advantage would be maintained in the absence of vision. The results showed that only the main effect hypotheses were supported, while the interaction hypothesis was not. In other words, throwing performance was superior when vision was available compared to occluded vision conditions, and both external and holistic focus of attention yielded better results than the internal focus condition.

First, the results demonstrated that javelin-throwing performance was significantly better under the vision condition compared to the no-vision condition. This finding is consistent with previous research indicating that visual information generally facilitates motor performance by supporting spatial orientation and movement calibration, particularly in novice performers [25,26,27,28].

In the javelin-throwing task, visual information is especially important during early skill acquisition, as novices must coordinate a complex sequence of approach, delivery, and release while orienting the body toward a far target. Vision supports perception–action coupling by providing continuous spatial information about the movement environment, which facilitates movement regulation and body orientation throughout task execution [31,32].

The availability of visual information may facilitate online regulation of the throwing movement. Previous research has shown that continuous visual information enables the sensorimotor system to support online control of goal-directed limb movements by detecting and correcting movement errors during execution [33]. Accordingly, visual availability likely resulted in better throwing performance in the present study, as reflected by the significant main effects of vision.

In addition to the main effects of vision, attentional focus instructions had a significant influence on performance outcomes. Specifically, both external and holistic focus instructions resulted in superior throwing performance compared to an internal focus of attention. These results are consistent with a large body of attentional focus literature demonstrating the performance disadvantages associated with directing attention toward body movements relative to focusing on movement effects or adopting a holistic attentional orientation [7,15,17,18,19,20,21,22].

The present findings extend previous attentional focus research to the context of javelin throwing: a whole-body ballistic skill that depends on the effective coordination of approach velocity, trunk rotation, and sequential segmental acceleration at release. In line with the prediction of the constrained action hypothesis [10], directing attention toward movement effects or outcomes may facilitate more automatic control and allow the motor system to self-organize around task outcomes rather than explicit movement mechanics, thereby potentially reducing the disruptive effects of conscious movement control. On the other hand, a holistic focus may facilitate coordinative structure formation and help preserve the integrity of the kinetic chain by reducing conscious control and self-monitoring of component movements [22]. In contrast, the internal focus may encourage more fragmented movement control, which could promote component-level monitoring and potentially interfere with intersegmental timing, leading to reduced movement efficiency [10].

Notably, no significant difference was observed between the external and holistic focus conditions, and both conditions outperformed the internal focus condition. Although this finding cannot demonstrate that both external and holistic attentional instructions influenced performance based on similar underlying control processes, it is possible that both approaches helped the throwers achieve more effective performance without explicitly attending to the components and movement details of their limbs. Holistic focus, by emphasizing attention to the overall movement pattern, may have reduced the need for conscious attention to movement mechanics, which is similar to the way external focus may influence performance. However, this interpretation remains speculative and should be directly examined in future related studies by assessing the underlying processes associated with different attentional focus strategies.

In addition, no statistically significant interaction between attentional focus and vision availability was detected, suggesting that the pattern of attentional focus effects did not differ appreciably between the two visual conditions. Some previous studies have reported that the performance benefits of an external focus are observed under both vision-available and vision-occluded conditions [26,27,28]. Consistent with these findings, our results slightly extend this prediction by demonstrating that, in novice performers, both holistic and external attentional foci outperform internal attention in javelin-throwing performance when averaged across the visual conditions.

Several limitations of the present study should be acknowledged. First, the relatively small sample of novice performers may limit the generalizability of the findings and may also have reduced the statistical power to detect an interaction between attentional focus and vision availability, as well as the robustness of the chi-square goodness-of-fit analysis examining participants’ preferences for attentional focus instructions. In addition, participants used the official competition javelins appropriate for their sex (600 g for females and 800 g for males); therefore, caution is warranted when generalizing the findings to other javelin weights. Consequently, these findings should be interpreted with appropriate caution. Future research should include larger samples of both sexes with varying levels of expertise, including skilled and elite javelin throwers.

Second, the study focused on performance outcomes without directly examining the underlying motor control mechanisms. Consequently, the proposed explanations regarding movement coordination and automatic control remain theoretical. Integrating electromyography (EMG) and kinematic analyses in future studies could provide a better understanding of how external and holistic attentional focus instructions influence muscle activation patterns and intersegmental coordination [7]. Third, because all experimental conditions were completed within a single testing session, learning, fatigue, or carry-over effects cannot be completely excluded despite the use of a counterbalanced design. Fourth, this study did not include retention or transfer tests; therefore, the findings are limited to immediate performance and cannot be generalized to motor learning.

Fifth, this study did not include a control condition; therefore, the relative benefits of the attentional focus instructions should be interpreted in comparison with one another rather than against a neutral baseline. Sixth, although the attentional focus instructions were adopted from previous research [7], they differed in their scope, temporal emphasis, and spatial references [34]. These instructional differences may have contributed to the observed effects in addition to attentional focus direction. Finally, visual information was manipulated dichotomously (vision vs. no vision), which may oversimplify the role of vision in attentional focus effects. Future research employing graded visual manipulations, such as stroboscopic eyewear [23], may provide a more comprehensive understanding of how different levels of visual information interact with attentional focus strategies to influence motor performance. Additionally, in the present study, only five valid throws were systematically recorded in each condition for the calculation of throwing distance, whereas invalid throws were repeated and their number was not recorded. Future studies should systematically record all attempts, including both valid and invalid throws, to allow for a more comprehensive assessment of the potential effects of visual information on throwing performance.

## 5. Conclusions

In summary, the present research highlights the importance of instructional strategies in motor performance, particularly in novice javelin throwers. The present findings indicate that visual information resulted in better throwing performance, while external and holistic attentional focus instructions produced superior performance compared with an internal focus. No statistically significant interaction between attentional focus and vision availability was observed. These findings have practical implications for coaches and instructors working with novice javelin throwers, suggesting that directing attention toward movement outcomes or overall movement coordination, rather than detailed body mechanics, may facilitate more effective motor performance.

## Figures and Tables

**Table 1:** Descriptive statistics for throwing distance and mean CV (%) across the six experimental conditions (*n* = 24).

Condition	Mean	SD	95% CI	Mean CV (%)
Vision—Internal	16.51	4.68	14.53–18.48	6.99
Vision—External	17.07	4.88	15.01–19.13	8.56
Vision—Holistic	17.50	5.57	15.15–19.85	7.49
No Vision—Internal	14.74	3.59	13.22–16.26	8.31
No Vision—External	15.96	4.39	14.10–17.81	9.52
No Vision—Holistic	16.28	5.41	14.00–18.57	8.45

## Data Availability

The datasets are available from the corresponding author on reasonable request.
